# Dopamine D3 receptor signaling alleviates mouse rheumatoid arthritis by promoting Toll-like receptor 4 degradation in mast cells

**DOI:** 10.1038/s41419-022-04695-y

**Published:** 2022-03-15

**Authors:** Biao Wang, Xueyi Li, Ming Li, Yan Geng, Na Wang, Yaofeng Jin, Wen Zhang, Ke Xu, Jing Wang, Li Tao, Simin Lai, Kunyi Wu, Jing Lei, Jing Wang, Ting Zhou, Ke Li, Yanjiong Chen, Li Xue

**Affiliations:** 1 Department of Immunology and Pathogenic Biology, College of Basic Medicine, Xi’an Jiaotong University Health Science Center, Xi’an, 710061 People’s Republic of China; 2 Department of Rheumatology, The Second Affiliated Hospital of Xi’an Jiaotong University, Xi’an, 710004 People’s Republic of China; 3 Department of Cardiovascular Surgery, The First Affiliated Hospital of Xi’an Jiaotong University, Xi’an, 710061 People’s Republic of China; 4 Department of Clinical Laboratory, The Second Affiliated Hospital of Xi’an Jiaotong University, Xi’an, 710004 People’s Republic of China; 5 Core Research Laboratory, The Second Affiliated Hospital of Xi’an Jiaotong University, Xi’an, 710004 People’s Republic of China; 6 Department of Pathology, The Second Affiliated Hospital of Xi’an Jiaotong University, Xi’an, 710004 People’s Republic of China; 7 Department of Pathology, Northwest Women’s and Children’s Hospital, Xi’an, 710061 People’s Republic of China; 8 Department of Joint Surgery, Xi’an Hong Hui Hospital, Xi’ an Jiaotong University Health Science Center, Xi’an, 710049 People’s Republic of China; 9 Department of Rheumatology, The First Affiliated Hospital of Xi’an Jiaotong University, Xi’an, 710061 People’s Republic of China; 10 National Local Joint Engineering Research Centre of Biodiagnostics and Biotherapy, The Second Affiliated Hospital of Xi’an Jiaotong University, Xi’an, 710004 People’s Republic of China

**Keywords:** Immunological disorders, Rheumatic diseases

## Abstract

Dopamine receptors are involved in several immunological diseases. We previously found that dopamine D3 receptor (D3R) on mast cells showed a high correlation with disease activity in patients with rheumatoid arthritis, but the mechanism remains largely elusive. In this study, a murine collagen-induced arthritis (CIA) model was employed in both DBA/1 mice and D3R knockout mice. Here, we revealed that D3R-deficient mice developed more severe arthritis than wild-type mice. D3R suppressed mast cell activation in vivo and in vitro via a Toll-like receptor 4 (TLR4)-dependent pathway. Importantly, D3R promoted LC3 conversion to accelerate ubiquitin-labeled TLR4 degradation. Mechanistically, D3R inhibited mTOR and AKT phosphorylation while enhancing AMPK phosphorylation in activated mast cells, which was followed by autophagy-dependent protein degradation of TLR4. In total, we found that D3R on mast cells alleviated inflammation in mouse rheumatoid arthritis through the mTOR/AKT/AMPK-LC3-ubiquitin-TLR4 signaling axis. These findings identify a protective function of D3R against excessive inflammation in mast cells, expanding significant insight into the pathogenesis of rheumatoid arthritis and providing a possible target for future treatment.

## Introduction

Rheumatoid arthritis (RA) is a chronic, progressive inflammatory autoimmune disease that shows distinct inflammation in joints with tissue swelling, damage, and malformation. RA patients typically present with symmetrical polyarthritis accompanied by other complications that impose great costs on both the individual and society. Hence, it is important to explore the pathogenesis of RA and its potent therapeutic target.

RA is caused by autoimmune responses, which involve both innate and adaptive immune responses, but the mechanism is not fully understood. Among multitudinous kinds of immune cells, mast cells (MCs) are attracting more attention in RA. They are present in the synovial membrane and have been implicated in contributing to the inflammatory response in RA [1]. A study by Lee et al. demonstrated that MCs may act as a cellular link between autoantibodies, soluble mediators, and other effector cells in inflammatory arthritis [2]. In a collagen-induced arthritis (CIA) model, MC depletion significantly reduced disease severity [3]. We previously found that the number of MCs in the synovial fluid was positively correlated with the disease severity of RA patients [4]. These data suggest that MCs play a crucial role in RA, while little is known about how the immune response of MCs is regulated in RA.

Dopamine (DA), an important catecholamine neurotransmitter, has been reported to regulate the activation of immune cells by binding to dopamine receptors (DRs) [5–7]. As reported, dopamine is detectable in the inflamed synovial tissue of RA patients [8]. Moreover, dopamine analogs have beneficial therapeutic effects in animal models and in patients with RA [9]. Notably, as a member of DRs, dopamine D3 receptor (D3R) displayed the highest affinity for dopamine. Our clinical research revealed that there is a negative correlation between D3R-positive MC numbers in the synovial fluid and disease severity in RA patients. Moreover, both in vivo and in vitro, we found that D3R directly inhibited the activation of MCs and suppressed proinflammatory cytokines upon challenge with lipopolysaccharide and methamphetamine (analogs of dopamine) [10]. This evidence indicates that D3R may play a role in the MC-involved pathogenesis of RA, while the underlying mechanism remains unclear.

We previously found that D3R blocked the TLR4-MAPK signaling cascade in activated MCs but how D3R regulates the TLR4 signaling pathway needs further study. In this study, we hypothesized that D3R might alleviate the inflammation of RA by inhibiting the expression of TLR4 on MCs. We aimed to determine the role of D3R in the regulation of TLR4 signaling and its related inflammatory cytokines in MCs, joint inflammation, and tissue destruction in a CIA model. Furthermore, we also investigated the degradation process of TLR4 by detecting ubiquitin and autophagy-related molecules.

## Material and methods

### Ethics statement

This study was approved by the Animal Ethics Committee of Xi’an Jiaotong University and carried out in accordance with their recommendations. All animal experimental procedures were performed under anesthesia that was induced and maintained with ketamine hydrochloride and xylazine, and all efforts were made to minimize the suffering of the animals.

### Mice

DBA/1 male mice (8 weeks) were obtained from Beijing Vital River Laboratory Animal Technology Co. Ltd. D3R-deficient (D3R^−/−^) mice were kind gifts from Professor Xu et al. (Department of Anesthesia and Critical Care, The University of Chicago). D3R^−/−^ mice have been previously described [11]. D3R^−/−^ mice and control wild-type (WT, D3R^+/+^) mice were bred under specific pathogen-free conditions in the same room of our animal facility. All mice used in this study were grouped randomly.

### CIA induction

As a well-validated mouse model of rheumatoid arthritis, CIA was used in our study. One hundred micrograms of chick type II collagen was emulsified in complete Freund’s adjuvant and intradermally injected into the mice at the base of the tail. Three weeks later, mice were given another intradermal injection of 100 μg chicken type II collagen, which was emulsified in incomplete Freund’s adjuvant [12, 13]. Clinical arthritis scores were assigned in each paw. 0 = normal, 1 = swelling and/or redness of paw or 1 digit, 2 = 2 joints involved, 3 = 3 joints involved, and 4 = severe arthritis of the entire paw and digit. The total score was based on all four paws, with a maximum score of 16 for each mouse. The incidence of arthritic paws was defined as the occurrence of inflamed paws with a clinical arthritis score of 2 or more by two independent observers in a blinded manner.

### Cell culture

The primary chondrocytes were isolated and cultured from mice as described previously [14]. Briefly, new-born mice (<5 d) were sacrificed after anesthesia and articular cartilage were isolated. Cartilage was cut into small pieces and digested for 45 min at 37 °C by 0.25% Trypsin. Cartilage pieces were then collected and placed in a cell dish with 0.2% collagenase II overnight in a thermal incubator under 5% CO_2_. The cells were used after being filtered by a cell strainer (Biologix, 70 μM).

p815 mast cells were purchased from the Kunming Institute of Zoology in the Chinese Academy of Sciences (Kunming, China). Bone marrow-derived mast cells (BMMCs) were derived from the femurs of 6–8-week-old D3R^−/−^ and WT mice as described previously[15]. These cells were cultured in RPMI 1640 medium supplemented with 10% FBS, 10 ng/ml IL-3, 10 ng/ml stem cell factor (SCF), 2 mM L-glutamine, 1 mM sodium pyruvate, 1 mM HEPES, 50 μM 2-mercaptoethanol, 100 U/ml penicillin, and 100 μg/ml streptomycin. IL-3 and SCF were purchased from Peprotech (Rocky Hill, NJ).

### Chemical molecule treatment

NGB2904 was dissolved in 50% polyethylene glycol 400 at a dose of 1 mg/kg body weight, and mice received intraperitoneal injections at intervals of 3 days after the second boost immunization [12]. The 7-OH schedule in mice was similar to that of NGB2904, while the dose was 0.5 mg/kg by subcutaneous injection [16]. NGB2904 (10 nM) and 7-OH (10 nM) were used to block or stimulate D3R, and 100 ng/ml LPS (Escherichia coli, serotype O55:B5) and 1 μM DA were used to prime mast cells [6, 8, 10, 17].

### Conditioned medium

Mast cells were exposed to 100 ng/ml LPS and 1 μM DA for 6 h and the medium was completely discarded. After washing three times, new medium was added to the dishes and incubated for another 24 h. The conditioned medium was collected after centrifugation at 2000 × *g* for 5 min.

### Mast cell inactivation

The mice were treated with the mast cell stabilizer ketotifen fumarate (Topscience, cat# 34580-14-8), which was added to *ad libitum* drinking water from Day 21 to Day 32. To obtain the final doses averaged 26.3 mg/kg/d to the mouse (25 g body weight, drank an average of 36.75 ml/d), ketotifen was diluted to 18.8 μg/ml in the drinking water. The practice is referred to Kathryn M. Lenz et al. [18].

### Protein silencing

Three pairs of short hairpin RNA (shRNA) sequences (shRNA1, shRNA2, and shRNA3) were designed according to the coding sequence of the *d3r* gene. They were cloned into the GV102 vector which showed ampicillin and neomycin resistance with a GFP label (Shanghai Genechem). The validity of the shRNAs was checked by sequencing before transfection. The scramble vector or the shRNA vectors were transfected into mast cells with Lipo6000 according to the manufacturer’s instructions (Shanghai Beyotime). After 48 h of transfection, the silencing was verified by immunoblotting.

### Cell viability assay

The standard 3-(4, 5-dimethylthiazol-2-yl)-2, 5-diphenyltetra-zolium bromide (MTT) assay was used to assess cell viability as previously described [19]. Briefly, mast cells were seeded in 96-well microtiter plates (6 × 10^4^ cells/well) for 2 h and then exposed to the various treatments for 24 h. After adding 20 μl MTT, which was diluted to 5 mg/ml in phosphate-buffered saline, the wells were incubated for another 4 h at 37 °C. DMSO was used to dissolve the metabolized MTT product, and a microplate reader was used to measure the optical density at 570 nm with a reference wavelength at 630 nm.

### ELISA

IL-1β, IL-6, and TNF-α cytokine levels in cell culture supernatants, joint tissues, and blood were detected using mouse ELISA kits (R&D Systems) after samples were processed according to the instructions.

### Immunoblotting analysis

Antibodies against D3R (#sc-136170), GAPDH (#sc-47724), p-p38 (#4511), p-ERK (#4370), p-JNK (#9255), p-c-jun (#3270), p-p65 (#3033), TLR4 (#14358), ubiquitin (#3936), LC3-I/II (#12741), mTOR (#2983), p-mTOR (#2971), Akt (#4691), p-Akt (#4060), p-AMPK (#4186) and AMPK (#sc-398861) were obtained from Santa Cruz Biotechnology (Santa Cruz, CA) or Cell Signaling Technology (Danvers, MA). The samples from cells and tissue were lysed using ice-cold RIPA lysis buffer (Solarbio, China) with 1% protease inhibitor cocktail (Roche, Switzerland) and 1% phosphorylase inhibitor cocktail (Roche, Switzerland). After the protein concentrations were quantified with a BCA protein assay kit (Beyotime, Jiangsu, China), the lysates were boiled for 10 min. The supernatants were collected, and 20 μg of each sample was loaded onto 10% SDS-polyacrylamide gel electrophoresis (PAGE) and electrophoresed for protein resolution. The proteins were then transferred to polyvinylidine difluoride membranes (0.22 μm, Thermo fisher, Rockford, IL) and blocked for 1 h at room temperature using 5% nonfat milk blocking buffer. The membranes were incubated for 2 h at room temperature with the appropriate primary antibodies diluted 1:1000 in 5% bovine serum albumin (BSA) immunoblotting antibody buffer. After washing (three times, 10 min once) with washing solution, the membranes were incubated for 60 min at room temperature with horseradish peroxidase-conjugated secondary antibody (Rockland Immunochemicals, Gilbertsville, PA) diluted 1:3000. Signals were visualized using an enhanced chemiluminescence detection kit (SuperSignal West Pico, Pierce). The results were evaluated with the gel image analysis software ImageJ 2.1.4.7.

### Confocal laser scanning microscopy

Cells were planted on slides and treated according to various experimental requirements. After washing three times with PBS, the slides were fixed in 4% PFA for 15 min and permeabilized with 0.2% Triton X-100 for 20 min. After that, the cells were blocked with 5% BSA for 30 min and incubated with specific primary antibodies at 1/500-1/1000 dilutions (according to the reagent manuals) for 8 h or overnight. Appropriate fluorophore-conjugated secondary antibodies were chosen to visualize specific antigens by incubating for another 1 h. Then, the slides were protected by coverslips and mounted with mounting medium. The cells were visualized and imaged by a LSM 510 Meta confocal laser scanning microscope (CLSM) (Carl Zeiss Micro Imaging, CA) [20, 21].

### Real-time quantitative PCR (qPCR)

Total RNA was extracted and purified from the cells and tissues using a TRIzol kit (Invitrogen, Carlsbad, CA, USA). Reverse transcription was performed to yield cDNA using a Prime Script TMRT reagent kit (Takara Bio Inc., Shiga, Japan). We chose SYBR Green II as the double-strand DNA-specific binding dye to complete the real-time qPCR on a Stratagene Mx 3005p Real-Time qPCR Detection System (Agilent Technologies, Santa Clara, CA, USA). The GAPDH gene was used as the loading control gene. All primers were synthesized by Tsingke Biotech (Xi’an, China). The forward and reverse primers used in the real-time qPCR are shown in Table S1. The real-time qPCR cycle was as follows: initial step at 95 °C for 30 s, 40 cycles at 95 °C for 5 s and 60 °C for 30 s, 1 cycle at 95 °C for 1 min, 55 °C for 30 s, and 95 °C for 30 s.

### Statistical analysis

Data are presented as the mean ± SEM. The sample size was estimated from preliminary experiments or from reports in the literature, and the numbers of samples are shown in the figure legends. After checking the normal distribution and the variance homogeneity of the data, two-tailed unpaired Student’s t-test was used to compare two groups, while one-way ANOVA (Tukey’s post hoc) was used for multiple group comparisons. P < 0.05 was accepted as a significant difference. All statistical analyses were performed by IBM SPSS Statistics 20.

## Results

### D3R deficiency augments RA severity in CIA mice

**Fig. 1:**
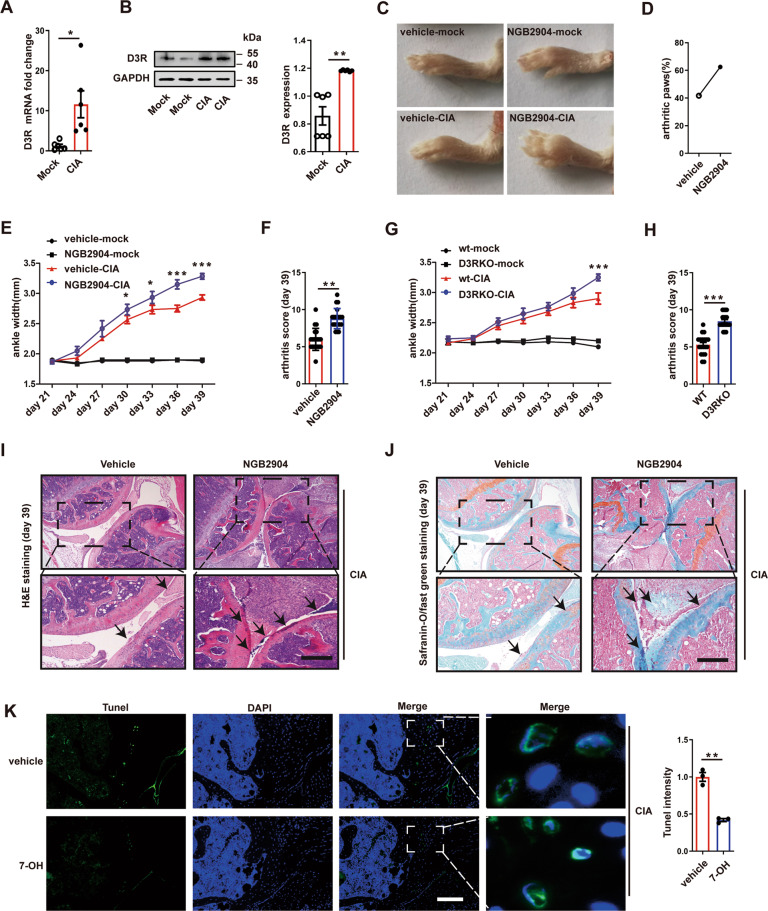
D3R alters RA severity in CIA mice. **A** and **B** After CIA induction in DBA/1 mice, ankle tissues were collected and lysed to test D3R expression by qPCR and western blotting (*n* = 6). **C** Mice received intraperitoneal injections of the D3R inhibitor NGB2904 at a dose of 1 mg/kg body weight at intervals of 3 days after the second boost immunization. The width of the ankle was assessed visually from the 21st day after the first immunization (0 days after the second boost immunization) at intervals of 3 days up to 39 days. The picture exhibited a significantly swelled paw on the 39th day. **D** We observed the ratio of arthritic paws, which was defined as the occurrence of inflamed paws with a clinical arthritis score of 2 or more (*n* = 20). **E** The ankle width from the 21st day to the 39th day (*n* = 20). **F** After NGB2904 treatment, the arthritis score in CIA mice was analyzed on the 39th day (*n* = 20). **G** After CIA induction in D3R knockout and WT mice, ankle width was measured from the 21st day to the 39th day (*n* = 19). **H** The arthritis score was analyzed on the 39th day (*n* = 19). **I** and **J** H&E and Saffron solid green staining were applied to observe synovial edema and hyperplasia, inflammatory infiltrates in the synovium, pannus formation, cartilage damage, and bone erosion in mouse joints after NGB2904 treatment. **K** A TUNEL assay was used to explore cell apoptosis in the joint (*n* = 6). All data are representative of the means ± SEM. Student’s t-test; *, *p* < 0.05; **, *p* < 0.01; ***, *p* < 0.001. Scale bar = 300 μm.

To investigate the role of D3R in RA, we detected the expression of D3R in DBA/1 mice after CIA induction. Real-time qPCR and western blotting showed that D3R expression was significantly increased in CIA mice (Fig. 1A, B). CIA induced characteristic swelling in paws, while the D3R inhibitor NGB2904 further aggravated tissue swelling in DBA/1 mice (Fig. 1C). Moreover, NGB2904 increased the percentage of arthritic paws (clinical arthritis score of 2 or more) on the 39th day (Fig. 1D). The ankle widths in mice were measured from the 21st day after the first immunization (0 day after the second boost immunization) at intervals of 3 days up to 39 days. The data showed that NGB2904 augmented the ankle width from the 30th day to the 39th day (Fig. 1E). After NGB2904 treatment, CIA mice developed higher arthritis scores on the 39th day (Fig. 1F). To further confirm the role of D3R in RA, we induced CIA in D3R knockout mice. D3R^−/−^ mice also developed more severe arthritis with significantly higher ankle width and arthritis score than WT mice on the 39th day (Fig. 1G, H). H&E and Saffron solid green staining showed that D3R^−^^/−^ CIA mice exhibited more severe synovial edema and hyperplasia, infiltration of inflammatory cells in the synovium, pannus formation, cartilage damage, and bone erosion than WT CIA mice (Fig. 1I, J). In addition, the TUNEL assay showed that the D3R agonist 7-OH significantly inhibited cell apoptosis in the joint (Fig. 1K). Together, these results demonstrate that D3R deficiency promotes RA severity in CIA mice.

### Mast cells are involved in D3R deficiency-induced excessive inflammation in the CIA model

**Fig. 2:**
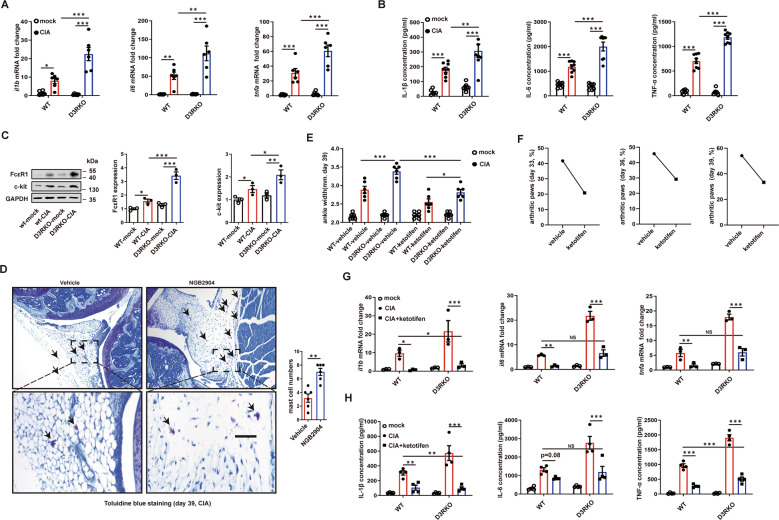
Mast cells are involved in D3R deficiency-induced excessive inflammation in the CIA model. **A** qPCR was used to examine the mRNA levels of *tnfa*, *il1b*, and *il6* in mouse knee joints after CIA induction (*n* = 6). **B** Serum cytokines were analyzed by ELISA in mice (*n* = 8). **C** FcεR1 and c-kit, markers of mast cells, were checked by western blotting in the knee joints of CIA mice (*n* = 4). **D** Toluidine blue staining was used to analyze mast cell numbers in mouse joints after CIA induction. Mast cell numbers were counted per visual field. **E** Ankle width was measured after treatment of CIA mice with the mast cell inhibitor ketotifen on Day 39. **F** After ketotifen treatment, the ratio of arthritic paws was observed on Days 33, 36, and 39. The ratio of arthritic paws was defined as the occurrence of inflamed paws with a clinical arthritis score of 2 or more (*n* = 20). **G** and **H** The mast cell inhibitor ketotifen (26.3 mg/kg/d) was used to abolish mast cell function, and then cytokines at both the mRNA and protein levels were tested by qPCR and western blotting (*n* = 4). All data are representative of the means ± SEM. Student’s t-test was used for (**D**), and one-way ANOVA (Tukey’s post hoc) was used for other panels; *, *p* < 0.05; **, *p* < 0.01; ***, *p* < 0.001. Scale bar = 30 μm.

Given the crucial role of inflammatory cytokines in RA, we also checked some related cytokines in hind paw tissues and serum. The data showed that, after CIA induction, D3R^−/−^ mice exhibited significantly higher *tnfa*, *il1b*, and *il6* mRNA levels in mouse knee joints, while there was no significant difference in the basal levels of these inflammatory cytokines between naïve D3R^−/−^ mice and WT mice (Fig. 2A). Serum cytokines (IL-1β, IL-6, TNF-α, IL-10, IL-4, and IL-13) were analyzed by ELISA, and it was shown that D3R^−/−^ mice had higher circulating levels of inflammatory cytokines than WT mice (Figs. 2B and S1). MCs are currently identified as important effector cells in the pathogenesis of RA. We previously found that D3R-positive MCs in the synovial fluid showed a declining trend with increasing disease activity in RA patients [4]. This finding indicated that D3R on MCs might play a regulatory role in the inflammatory process of RA. To prove this hypothesis, we first examined the markers of MCs in the knee joints of CIA mice. Western blotting showed that the expression of the MC markers FcεR1 and c-kit was significantly increased in CIA mice, while D3R deficiency augmented the contents of both markers (Fig. 2C). Moreover, Toluidine blue staining visually showed that more MCs were present in the joints from D3R^−/−^ mice than those in WT mice (Fig. 2D). At the same time, the ankle widths of D3R^−/−^ mice were declined after they were treated with ketotifen (Fig. 2E). In addition, the incidence of CIA was distinctly restrained by ketotifen on Days 33, 36, and 39 (Fig. 2F). To further explore the role of MCs in D3R altered CIA cytokines, we used the MC inhibitor ketotifen to abolish the function of MCs. The data showed that ketotifen significantly inhibited D3R deficiency-augmented cytokines at both the mRNA and protein levels (Fig. 2G, H). These data suggest that MCs might play a crucial role in D3R regulated inflammation in CIA.

### D3R restores chondrocyte viability by restricting excessive inflammation in MCs

**Fig. 3:**
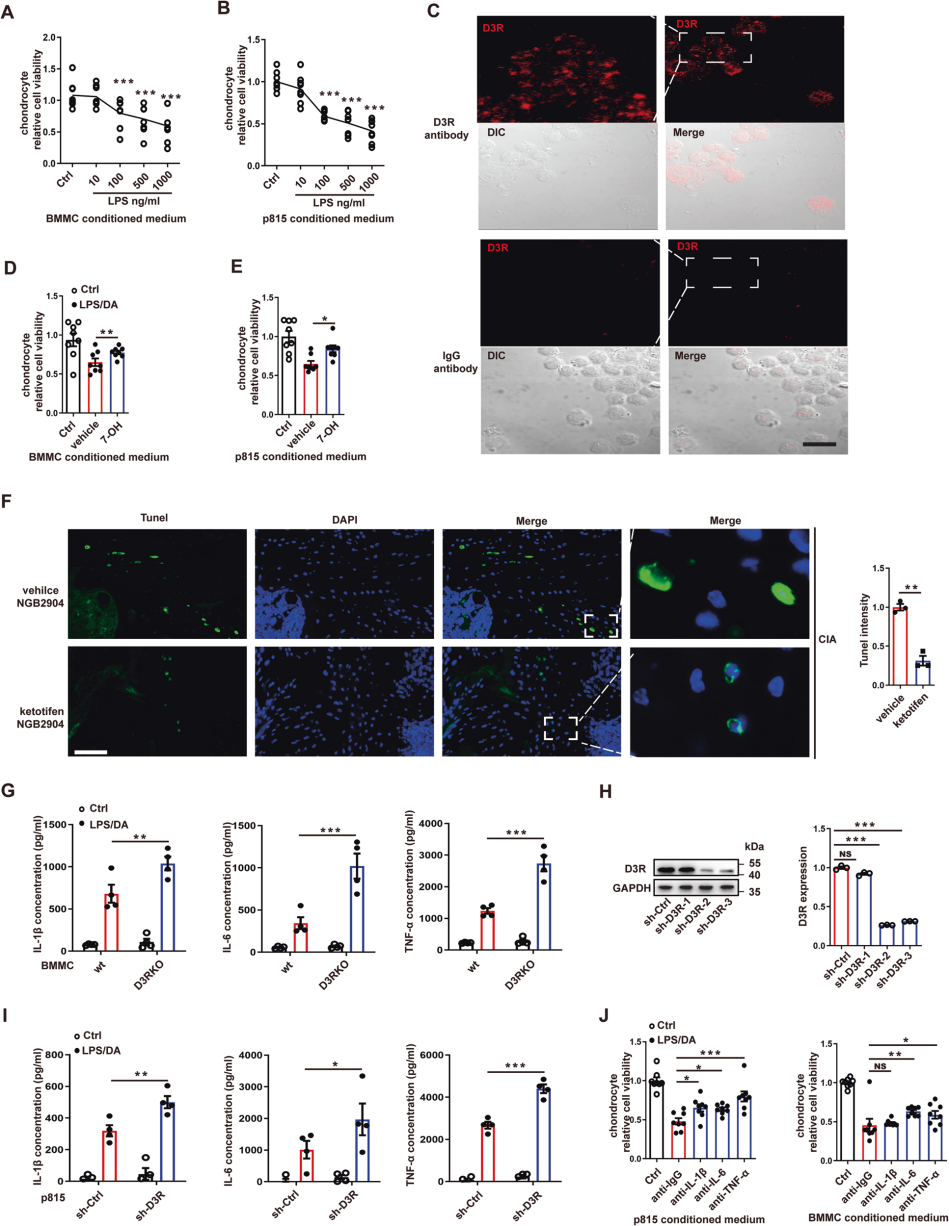
Mast cells mediate the function of D3R in the CIA model via altered inflammation. **A** and **B** After mast cells were activated by lipopolysaccharide and dopamine (DA, 1 μM) at various doses, the culture medium was collected and added to chondrocyte bedded 96-well plates. The changes in primary chondrocyte cell activity were checked by MTT assay. **C** Laser confocal microscopy was used to observe the expression of D3R on mast cells. **D** and **E** An MTT assay for chondrocyte cells was performed again after treatment with the D3R agonist 7-OH in mast cells. **F** After ketotifen and NGB2904 treatment, TUNEL staining was used to check cell apoptosis in the joint on Day 39. **G** Cytokines were tested by ELISA after BMMCs were exposed to LPS and DA. **H** Three shRNA plasmids were constructed and transfected into p815 cells. **I** Cytokines were tested by ELISA after shRNA-treated p815 cells were exposed to LPS and DA. **J** Mast cells were treated with protein A/G magnetic beads pre-cross-linked with IL-1β, IL-6, and TNF-α antibodies, and then chondrocyte viability was tested by MTT assay after the addition of mast cell medium after the beads were discarded. LPS (100 ng/ml) and DA (1 μM) were used to activate the mast cells. All data are representative of the means ± SEM. Student’s t-test was used for (**F**), and one-way ANOVA (Tukey’s post hoc) was used for other panels, *n* = 3–8; *, *p* < 0.05; **, *p* < 0.01; ***, *p* < 0.001. Scale bar = 30 μm (C) or 300 μm (**H**).

To further explore the actual function of D3R on MCs in RA, we used lipopolysaccharide to activate MCs, and then collected the medium of these MCs to treat primary chondrocytes. The data showed that activated MCs significantly reduced the viability of chondrocytes (Fig. 3A, B). After D3R was proven to be expressed on MCs using laser confocal microscopy (Fig. 3C), we applied the D3R agonist 7-OH to investigate the role of D3R in this process. We found that the medium collected from 7-OH-treated MCs restored the viability of chondrocytes (Fig. 3D, E). Furthermore, TUNEL staining showed that ketotifen significantly mitigated cell apoptosis in the joint (Fig. 3F). In vitro, D3R deficiency strongly augmented the expression of IL-1β, IL-6, and TNF-α in BMMCs (Fig. 3G). After choosing efficient D3R shRNA, we knocked down D3R in the p815 mast cell line (Fig. 3H). D3R shRNA showed similar results for inflammatory cytokines in p815 cells compared with D3R-deficient BMMCs (Fig. 3I). Moreover, we added antibodies against IL-1β, IL-6, and TNF-α to the medium of MCs and found that the addition of antibodies could improve the viability of chondrocytes while anti-IgG still greatly impaired the activity (Fig. 3J). Together, these results demonstrated that D3R regulated joint damage via alterations in proinflammatory cytokines secreted by mast cells.

### TLR4-MAPK signaling is involved in D3R-altered mast cell inflammation

**Fig. 4:**
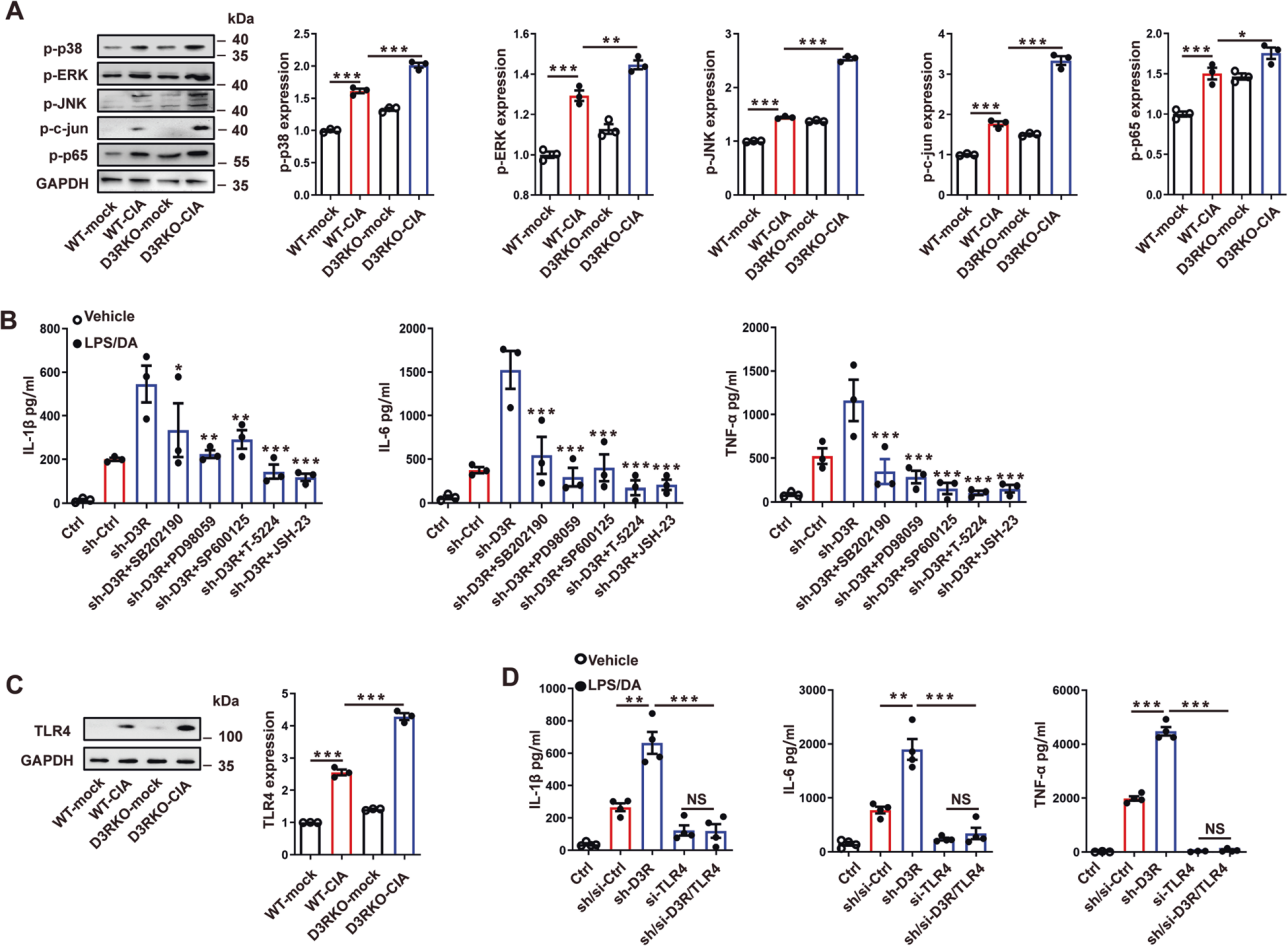
TLR4-MAPK signaling is involved in D3R-altered mast cell inflammation. **A** Changes in the signaling of p38, ERK, and JNK, as well as their downstream molecules AP-1 (c-jun) and NF-κB (p65), were tested by western blotting in a mouse CIA model. **B** ELISA was used to check the cytokines after mast cells were treated with signaling inhibitors to block their function. p38 inhibitor SB202190 20 μM, ERK inhibitor PD98059 10 μM, JNK inhibitor SP600125 20 μM, c-jun inhibitor T-5224 30 μM and p65 inhibitor JSH-23 10 μM. **C** Changes in TLR4 in the mouse knee joint were observed by western blotting. **D** Both D3R and TLR4 in p815 mast cells were silenced, and cytokines were measured. All data are representative of the means ± SEM. One-way ANOVA (Tukey’s post hoc), *n* = 3–4; *, *p* < 0.05; **, *p* < 0.01; ***, *p* < 0.001.

We previously found that MAPK signaling molecules played a crucial role in activated mast cells after methamphetamine exposure. We wondered whether MAPKs were also involved in the D3R-regulated inflammatory response of MCs in the pathogenesis of CIA. The data showed that CIA significantly increased the phosphorylation levels of p38, ERK, and JNK, as well as their downstream molecules AP-1 (c-jun) and NF-κB (p65) (Fig. 4A). At the same time, D3R^−/−^ mice exhibited higher phosphorylation levels of MAPKs than WT control mice (Fig. 4A). SB202190, a p38 inhibitor, significantly restricted the production of IL-1β, IL-6, and TNF-α (Fig. 4B). Consistently, the same results were shown when we used the ERK inhibitor PD98059, JNK inhibitor SP600125, c-jun inhibitor T-5224, and p65 inhibitor JSH-23, indicating the involvement of all these MAPK signaling molecules in the process and hinting at the possible participation of upstream molecules of MAPK signaling in the regulation (Fig. 4B). Hence, we checked the changes in TLR4, which triggered the phosphorylation of MAPKs upon activation. We found that CIA mice showed high expression of TLR4 in the knee joint, while the level of TLR4 was augmented in D3R^−/−^ mice (Fig. 4C). In addition, TLR4 knockdown abolished D3R-shRNA-induced excessive production of proinflammatory cytokines in p815 MCs (Fig. 4D).

### D3R promotes TLR4 degradation in mast cells in a ubiquitin- and LC3-II-dependent manner

**Fig. 5:**
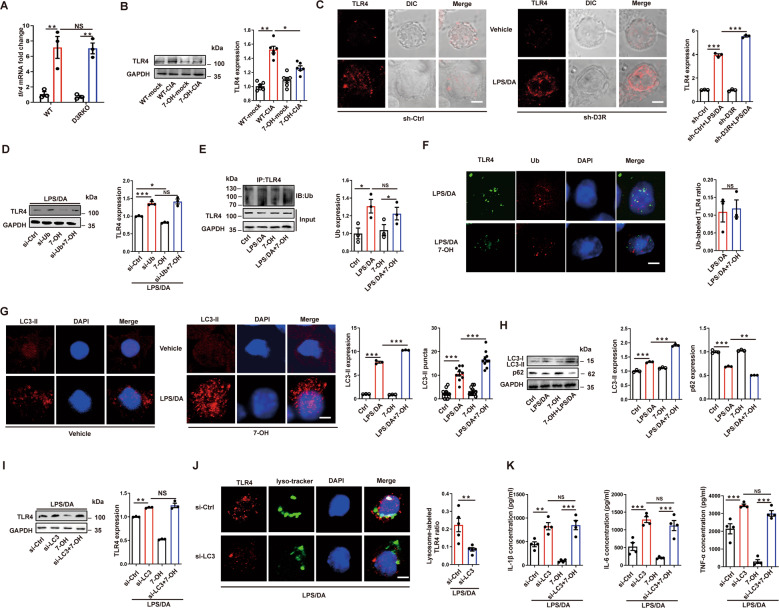
D3R promotes TLR4 degradation in mast cells in a ubiquitin- and LC3-II-dependent manner. **A** *trl4* mRNA levels in joints were examined after CIA induction (*n* = 3). **B** TLR4 expression was checked after mice were treated with the D3R agonist 7-OH (*n* = 6). **C** TLR4 staining was exhibited by laser confocal microscopy images in p815 mast cells after D3R knockdown and LPS/DA exposure. The mean fluorescence intensity was measured by ImageJ software (*n* = 3). **D** TLR4 expression was checked after ubiquitin (Ub) knockdown and 7-OH treatment upon LPS/DA exposure in p815 mast cells (*n* = 3). **E** Ubiquitin content was examined after pulling down TLR4 in p815 mast cells (*n* = 3). **F** The colocalization of TLR4 with ubiquitin was checked after 7-OH treatment in p815 mast cells (*n* = 3). **G** and **H** LC3-II puncta (*n* = 10) and LC3-II and p62 expression (*n* = 3) were observed by laser confocal microscopy images or western blotting. Mean fluorescence intensity was used to represent the expression of LC3-II in p815 mast cells. **I** TLR4 expression was examined after LC3-II knockdown in p815 mast cells. **J** The colocalization of TLR4 with lysosomes was checked after LC3-II knockdown in p815 mast cells (*n* = 5). **K** ELISA revealed proinflammatory cytokines in activated p815 mast cells after LC3-II knockdown and 7-OH treatment (*n* = 4). All data are representative of the means ± SEM. Student’s t-test was used for (**F**), and one-way ANOVA (Tukey’s post hoc) was used for other panels; *, *p* < 0.05; **, *p* < 0.01; ***, *p* < 0.001. Scale bar = 5 μm.

We previously proved that D3R inhibited the expression of TLR4 in activated MCs, but the mechanism was not clear. First, we wondered whether D3R impaired TLR4 expression by inhibiting transcription of the *tlr4* gene. The data showed that D3R deficiency did not change the level of *tlr4* mRNA in the CIA model, indicating that D3R has no effect on *tlr4* transcription (Fig. 5A). Hence, given the augmented expression of TLR4 in the CIA model (Fig. 4C), we wondered whether D3R could promote the degradation of TLR4. To confirm this hypothesis, we subcutaneously injected the D3R agonist 7-OH into CIA mice and found that the expression of TLR4 in the mouse knee joint was significantly decreased (Fig. 5B). In contrast, laser confocal microscopy imaging showed that D3R shRNA distinctly increased TLR4 staining in activated p815 MCs (Fig. 5C).

Given that D3R deficiency did not change the mRNA level of TLR4 but had an effect on its protein level and that it was previously reported that activated TLR4 could be translocated into lysosomes for degradation, we next investigated whether D3R could downregulate the protein level of TLR4 by promoting the degradation of TLR4 [22]. Ubiquitin and LC3-II are regarded as two important molecules in the process of handling and translocating superfluous or overactivated proteins into lysosomes. Our results showed that ubiquitin siRNA significantly increased the expression of TLR4 in activated p815 MCs and fully abolished the function of the D3R agonist 7-OH, indicating the critical role of ubiquitin in TLR4 degradation (Fig. 5D). We further pulled down TLR4 to determine whether D3R could alter the binding of ubiquitin with TLR4. We found that there was no difference in the level of ubiquitin combined with TLR4 between the two groups with or without 7-OH treatment (Fig. 5E). In addition, laser confocal microscopy images also exhibited the relatively same ratio of ubiquitin to TLR4 between the two groups with or without 7-OH treatment (Fig. 5F).

Then, we examined whether LC3-II was involved in D3R-mediated regulation of TLR4 degradation, while LC3-II was regarded as the bridge between lysosomes and cargos that need to be digested. The results from both laser confocal microscopy and western blotting showed that puncta or the expression of LC3-II increased in activated p815 MCs, while 7-OH could further significantly promote the level of LC3-II (Fig. 5G, H). In addition, p62 was downregulated at the same time, indicating that 7-OH could alter autophagic flux in activated MCs (Fig. 5H). We further used LC3 siRNA to silence LC3 and found that LC3 siRNA treatment increased TLR4 expression (Fig. 5I). More importantly, 7-OH failed to reverse the increase in TLR4 which was induced by LC3 silencing (Fig. 5I). Consistently, silencing LC3 significantly limited the colocalization of TLR4 with lysosomes (Fig. 5J). In addition, LC3 and ubiquitin silencing remarkably blocked the 7-OH-induced reduction in proinflammatory cytokines in activated MCs (Figs. 5K and S3). Taken together, these results suggest that D3R promotes TLR4 degradation in MCs in a LC3-II-dependent manner and that LC3-II is a potential target in the TLR4 degradation process.

### D3R inactivates mTOR and AKT while promoting AMPK activation to initiate TLR4 degradation

**Fig. 6:**
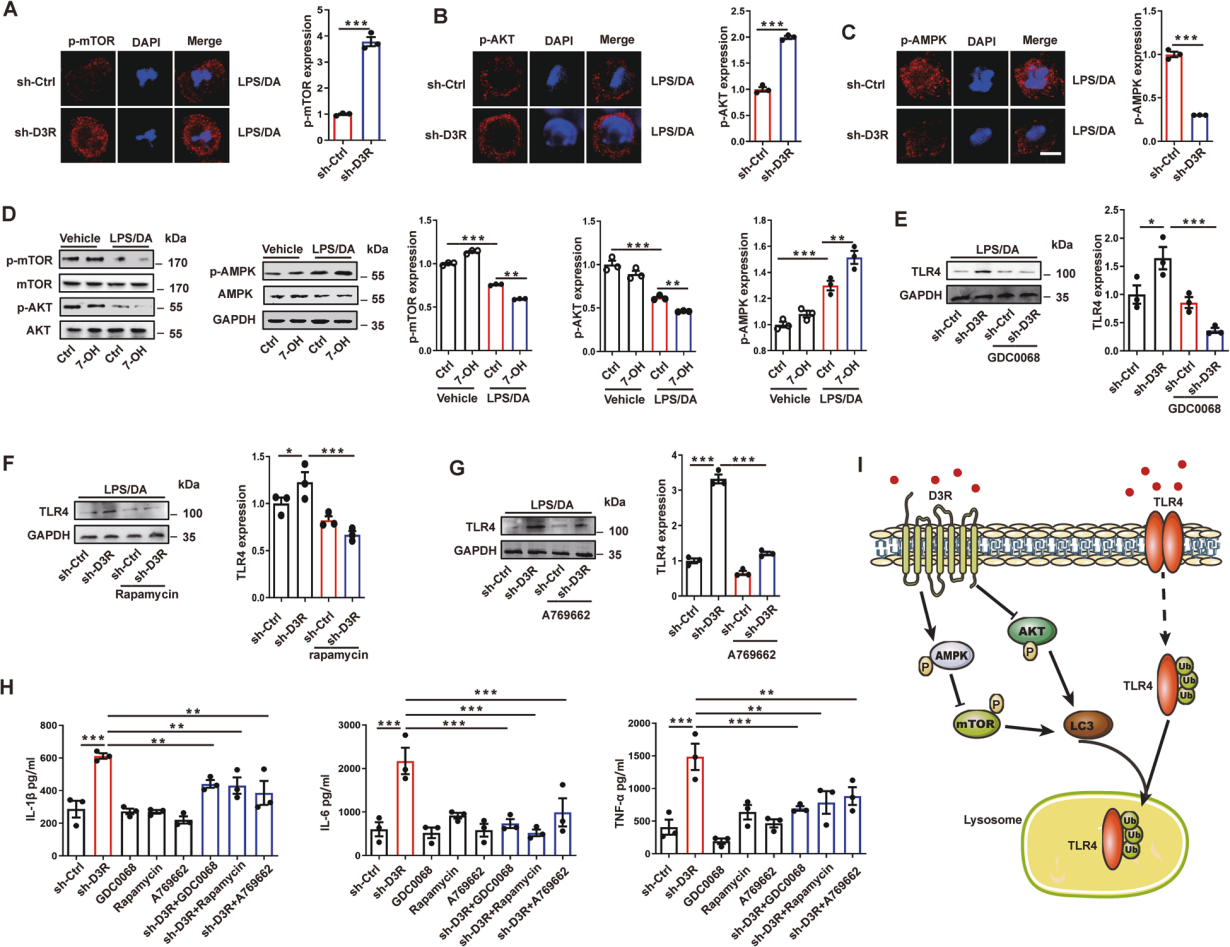
D3R inactivates mTOR and AKT while promoting AMPK activation to initiate TLR4 degradation. **A**–**C** After D3R knockdown, phosphorylated mTOR, AKT, and AMPK content was checked by laser confocal microscopy in p815 mast cells. Mean fluorescence intensity was used to represent the expression. **D** Phosphorylated mTOR, AKT, and AMPK were checked by western blotting after 7-OH treatment. **E**–**G** TLR4 expression was checked by western blotting after the p815 mast cells were treated with the AKT antagonist GDC0068 (1 μM), mTOR inhibitor rapamycin (50 μM), and AMPK agonist A769662 (100 μM) after D3R knockdown. **H** The cytokines in activated p815 mast cells were measured after above the antagonists and agonist treatment. **I** Schematic diagram showing how TLR4 degradation is regulated by D3R and potent signaling pathways. Student’s t-test was used for (**F**) and one-way ANOVA (Tukey’s post hoc) was used for other panels; *, *p* < 0.05; **, *p* < 0.01; ***, *p* < 0.001, *n* = 3. Scale bar = 5 μm.

Another question was how D3R triggered the LC3-II-dependent degradation of TLR4. We examined the autophagy initiating factors, such as mTOR, AKT, and AMPK, which are upstream molecules that recruit LC3-II to label cargos. We found that D3R knockdown elevated the levels of phosphorylated mTOR and AKT but inhibited AMPK phosphorylation in MCs (Fig. 6A–C). In contrast, the D3R agonist 7-OH inhibited the phosphorylation of mTOR and AKT while promoting AMPK phosphorylation (Fig. 6D). To further confirm the role of these autophagy initiating factors in D3R-induced degradation of TLR4, we applied the AKT antagonist GDC0068, mTOR inhibitor rapamycin and AMPK agonist A769662. The data showed that all the chemical reagents eradicated D3R knockdown-induced excessive TLR4 expression (Fig. 6E–G). To verify these results, we tested the regulatory role of mTOR, AKT, and AMPK in proinflammatory cytokines in activated MCs. GDC0068, rapamycin, and A769662 dramatically reduced the production of IL-1β, IL-6, and TNF-α cytokines in MCs treated with D3R shRNA (Fig. 6H). These data suggest that mTOR, AKT, and AMPK mediate D3R-induced TLR4 degradation.

## Discussion

Dopamine (DA) is a neurotransmitter in the central and peripheral nervous systems and is crucially important in cognition, motivation, and movement [23]. Accumulating evidence has indicated a crucial role of dopamine signaling in the regulation of inflammatory responses. In the central nervous system, we previously found that dopamine signaling altered cytokines, including IL-1β, IL-6, TNF-α, and nitric oxide, in LPS-challenged brains and microglia in vitro [17, 19, 24]. In the peripheral immune system, dopamine signaling greatly changed the immune response in macrophages and mast cells [10, 25]. In addition, more evidence from patients and animal models shows that altered dopaminergic activation is associated with the physiopathology of autoimmune diseases, such as RA [4, 26, 27]. Consistently, we previously uncovered a positive association between D3R expression on MCs and disease activity in patients with RA [4]. In the present study, we reported the critical role of D3R in RA by altering the inflammatory response of MCs.

Many immune cells have been demonstrated to be able to express D3R, and increasing attention has been given to the role of D3R in RA [9, 28]. It was reported that a D3R agonist limited the levels of proinflammatory cytokines and the degree of arthritis pathology [26]. It was also reported that the expression of D3R on peripheral B cells was increased in RA patients [7]. All these findings suggested that D3R on immune cells may be involved in the pathogenesis of RA. In this study, the D3R inhibitor NGB2904 aggravated tissue swelling and ankle width in CIA mice. D3R^−/−^ mice developed more severe arthritis with significantly greater ankle width and higher levels of proinflammatory cytokines than WT mice. Moreover, our results showed that D3R^−^^/−^ mice exhibited more severe synovial edema, and more inflammatory infiltrates and cell apoptosis in the joints of a CIA model. In addition, similar results were also reported by Nakano et al. who showed that a D3R antagonist obviously augments cartilage destruction in a human RA/SCID mouse chimera model [8]. As reported, dopamine is detectable in the inflamed synovial tissue of RA patients [8]. To mimic the real RA pathological conditions, dopamine is combined with 7-OH in our experiments in vitro. However, this design makes interpretation complicated, and more exploration is needed to confirm dopamine and D3R function. Together, these results demonstrate that D3R deficiency promotes the progression of RA, while the underlying mechanisms require further investigation.

MCs have been demonstrated to act as a cellular link between autoantibodies, soluble mediators, and other effector cells in inflammatory arthritis, demonstrating that mast cell-deficient mice are resistant to the development and pathogenesis of inflammatory arthritis [2]. This finding was also proven by other study groups via mast cell ablation [29]. Here, we found that the MC markers FcεR1 and c-kit were significantly upregulated in CIA mice, while D3R deficiency further augmented the expression of both markers. Moreover, our results showed that the number of MCs in the joints of D3R^−/−^ CIA mice was distinctly higher than that of WT mice. A previous study reported that the aggregation of MCs in the synovium was closely associated with the pathogenesis of RA [30]. Moreover, we also found that the MC inhibitor ketotifen reversed D3R deficiency induced excessive cytokines in CIA mice. All these findings suggest that MCs are involved in D3R-regulated inflammatory arthritis. In addition, given the complexity of RA pathophysiology and the interaction among all the immune cells, we can’t rule out other immune cells in D3R function, though ketotifen is usually used as an effective inhibitor of mast cells. There is one possibility that the changes of IL-1β, IL-6, TNF-α, IL-4, and IL-13 in D3RKO mice is a result of the interaction between mast cells and other cell types. However, this possibility needs more evidence in future work.

TLR4 is critical in innate and acquired immunity, including the immune response of MCs [31, 32]. TLR4 is highly expressed in synovial tissue from individuals with RA [33]. Therefore, TLR4 is attracting more attention in the field of autoimmune diseases. After TLR4 activation, MCs secrete many cytokines, such as IL-4, IL-6, IL-13, TNF-α, and CCL-5 [34]. In addition, mice with gene deletions of *tlr4* or loss-of-function mutations were protected from experimental arthritis [35]. Furthermore, inhibitors of TLR4 improved symptoms of patients with rheumatoid arthritis in a preliminary phase 1 trial [36]. In our previous study, we found that D3R inhibited the expression of TLR4 and its downstream signaling molecules after MCs were exposed to LPS, a ligand of TLR4, and methamphetamine, a reuptake inhibitor of dopamine [15]. Here, we also found that D3R deficiency remarkably upregulated TLR4 expression and the phosphorylation of MAPKs (p38, ERK, and JNK), AP-1 (c-jun), and NF-κB. Moreover, inhibitors of these signaling molecules significantly inhibited the production of proinflammatory cytokines in activated MCs. These findings suggest that TLR4 is involved in D3R-mediated regulation of the inflammatory response in RA.

We found that D3R regulated the expression of TLR4 on MCs, but little is known about the underlying mechanism. First, we checked the gene transcription of *tlr4* and found that D3R deficiency did not change the *trl4* mRNA level in the joints of CIA mice. This finding suggests that *tlr4* gene transcription is not the target of D3R-mediated regulation. It was reported that TLR4-induced immune responses were negatively regulated by the translocation of TLR4 into lysosomes for its degradation [22]. In this study, we found that the downregulation of both ubiquitin and LC3, which are two critical molecules in protein degradation, could increase the protein level of TLR4 and block the 7-OH-induced anti-inflammatory response in MCs. This evidence suggests that D3R may promote the degradation of TLR4 via the help of ubiquitin and LC3 in activated MCs. In addition, the proteasome-related degradation pathway is also important for ubiquitinated proteins in the cytosol. Although D3R did not alter ubiquitin expression or ubiquitin-labeled TLR4 ratio in activated mast cells in our results, we can’t exclude the possibility that the proteasome is also involved in D3R-mediated TLR4 degradation. Hence, the role of the proteasome needs more exploration in the following work.

Moreover, we found that some autophagy-initiating molecules, such as mTOR, AKT, and AMPK, were involved in the degradation of TLR4 and the production of proinflammatory cytokines in activated MCs. D3R altered their phosphorylation levels, which directly regulated the recruitment of LC3-II to cargos that need to be translocated to lysosomes. It has been reported that D3R activates AMPK followed by inhibitory phosphorylation of mTOR, which eventually contributes to the clearance of misfolded proteins [37]. A recent study also revealed that D3R triggered autophagy induction via the inhibition of mTOR activity and changed mTOR localization in ammonia-treated HEK293T cells [38]. In addition, D3R expressed in DA neurons could regulate the antidepressant ketamine-induced activation of mTOR signaling [39, 40]. In human iPSC-derived DA neurons, D3R-dependent recruitment of mTOR signaling increases structural plasticity [41]. All the evidence suggests that mTOR signaling mediates D3R function, and moreover, the D3R-mTOR axis plays a critical role in a good deal of diseases and shows good application prospects in therapy.

## Conclusions

In conclusion, we proved that D3R inhibited mast cell inflammation that alleviated mouse rheumatoid arthritis by promoting TLR4 degradation. The mTOR/AKT/AMPK-LC3-ubiquitin signaling axis takes part in this process that bridges mast cell activation and TLR4 degradation in rheumatoid arthritis. These findings identify a protective function of D3R against excessive inflammation in MCs, expanding significant insight into the pathogenesis of RA and providing a possible target for the future of treatment.

## Supplementary information

checklist

SUPPLEMENTAL MATERIAL INFORMATION

Figure S1

Figure S2

Figure S3

Figure S4

Table S1

## Data Availability

The uncropped Western blots used in this study are included in Supplementary Fig. 4. The rest datasets used or analyzed in the current study are available upon reasonable request from the corresponding author.

## References

[1] Rivellese F, Nerviani A, Rossi FW, Marone G, Matucci-Cerinic M, de Paulis A (2017). Mast cells in rheumatoid arthritis: Friends or foes?. Autoimmun Rev.

[2] Lee DM, Friend DS, Gurish MF, Benoist C, Mathis D, Brenner MB (2002). Mast cells: A cellular link between autoantibodies and inflammatory arthritis. Science..

[3] Schubert N, Dudeck J, Liu P, Karutz A, Speier S, Maurer M (2015). Mast cell promotion of T cell-driven antigen-induced arthritis despite being dispensable for antibody-induced arthritis in which T cells are bypassed. Arthritis Rheumatol.

[4] Xue L, Li X, Chen Q, He J, Dong Y, Wang J (2018). Associations between D3R expression in synovial mast cells and disease activity and oxidant status in patients with rheumatoid arthritis. Clin Rheumatol.

[5] Sarkar C, Basu B, Chakroborty D, Dasgupta PS, Basu S (2010). The immunoregulatory role of dopamine: An update. Brain Behav Immun.

[6] Yan Y, Jiang W, Liu L, Wang X, Ding C, Tian Z (2015). Dopamine controls systemic inflammation through inhibition of NLRP3 inflammasome. Cell..

[7] Shao W, Zhang S-z, Tang M, Zhang X-h, Zhou Z, Yin Y-q (2012). Suppression of neuroinflammation by astrocytic dopamine D2 receptors via αB-crystallin. Nature..

[8] Nakano K, Yamaoka K, Hanami K, Saito K, Sasaguri Y, Yanagihara N (2011). Dopamine induces IL-6-dependent IL-17 production via D1-like receptor on CD4 naive T cells and D1-like receptor antagonist SCH-23390 inhibits cartilage destruction in a human rheumatoid arthritis/SCID mouse chimera model. J Immunol.

[9] Pacheco R, Contreras F, Zouali M (2014). The dopaminergic system in autoimmune diseases. Front Immunol.

[10] Xue L, Li X, Ren H-X, Wu F, Li M, Wang B (2015). The dopamine D3 receptor regulates the effects of methamphetamine on LPS-induced cytokine production in murine mast cells. Immunobiology..

[11] Zhu J, Chen Y, Zhao N, Cao G, Dang Y, Han W (2012). Distinct roles of dopamine D3 receptors in modulating methamphetamine-induced behavioral sensitization and ultrastructural plasticity in the shell of the nucleus accumbens. J Neurosci Res.

[12] Pritchard LM, Newman AH, McNamara RK, Logue AD, Taylor B, Welge JA (2007). The dopamine D3 receptor antagonist NGB 2904 increases spontaneous and amphetamine-stimulated locomotion. Pharm Biochem Behav.

[13] Wang N, Wu W, Qiang C, Ma N, Wu K, Liu D (2021). Protective role for collectin‐11 in rheumatoid arthritis in mice. Arthritis Rheumatol.

[14] Gosset M, Berenbaum F, Thirion S, Jacques C (2008). Primary culture and phenotyping of murine chondrocytes. Nat Protoc.

[15] Xue L, Geng Y, Li M, Jin Y-F, Ren H-X, Li X (2016). The effects of D3R on TLR4 signaling involved in the regulation of METH-mediated mast cells activation. Int Immunopharmacol.

[16] Lao CL, Kuo YH, Hsieh YT, Chen JC (2013). Intranasal and subcutaneous administration of dopamine D3 receptor agonists functionally restores nigrostriatal dopamine in MPTP-treated mice. Neurotox Res.

[17] Wang B, Chen T, Li G, Jia Y, Wang J, Xue L (2019). Dopamine alters lipopolysaccharide-induced nitric oxide production in microglial cells via activation of D1-like receptors. Neurochem Res.

[18] Lenz KM, Pickett LA, Wright CL (2018). Mast cells in the developing brain determine adult sexual behavior. J Neurosci.

[19] Wang B, Chen T, Wang J, Jia Y, Ren H, Wu F (2018). Methamphetamine modulates the production of interleukin-6 and tumor necrosis factor-alpha via the cAMP/PKA/CREB signaling pathway in lipopolysaccharide-activated microglia. Int Immunopharmacol.

[20] Li Y, Gan CP, Zhang S, Zhou XK, Li XF, Wei YQ (2014). FIP200 is involved in murine pseudomonas infection by regulating HMGB1 intracellular translocation. Cell Physiol Biochem.

[21] Li R, Fang L, Pu Q, Lin P, Hoggarth A, Huang H (2016). Lyn prevents aberrant inflammatory responses to Pseudomonas infection in mammalian systems by repressing a SHIP-1-associated signaling cluster. Signal Transduct Target Ther.

[22] Wang Y, Chen T, Han C, He D, Cao X (2007). Lysosome-associated small Rab GTPase Rab7b negatively regulates TLR4 signaling in macrophages by promoting lysosomal degradation of TLR4. Blood..

[23] Romanov RA, Zeisel A, Bakker J, Girach F, Hellysaz A, Tomer R (2017). Molecular interrogation of hypothalamic organization reveals distinct dopamine neuronal subtypes. Nat Neurosci.

[24] Wang B, Chen T, Xue L, Wang J, Jia Y, Li G (2019). Methamphetamine exacerbates neuroinflammatory response to lipopolysaccharide by activating dopamine D1-like receptors. Int Immunopharmacol.

[25] Li X, Wu F, Xue L, Wang B, Li J, Chen Y (2018). Methamphetamine causes neurotoxicity by promoting polarization of macrophages and inflammatory response. Hum Exp Toxicol.

[26] Lu JH, Liu YQ, Deng QW, Peng YP, Qiu YH (2015). Dopamine D2 receptor is involved in alleviation of type II collagen-induced arthritis in mice. Biomed Res Int.

[27] van Nie L, Salinas-Tejedor L, Dychus N, Fasbender F, Hülser ML, Cutolo M (2020). Dopamine induces in vitro migration of synovial fibroblast from patients with rheumatoid arthritis. Sci Rep.

[28] Levite M (2016). Dopamine and T cells: Dopamine receptors and potent effects on T cells, dopamine production in T cells, and abnormalities in the dopaminergic system in T cells in autoimmune, neurological, and psychiatric diseases. Acta Physiol.

[29] Feyerabend T, Weiser A, Tietz A, Stassen M, Harris N, Kopf M (2011). Cre-mediated cell ablation contests mast cell contribution in models of antibody- and T cell-mediated autoimmunity. Immunity..

[30] Eklund KK (2010). Mast cells in the pathogenesis of rheumatic diseases and as potential targets for anti-rheumatic therapy. Immunol Rev.

[31] Akira S, Takeda K, Kaisho T (2001). Toll-like receptors: Critical proteins linking innate and acquired immunity. Nat Immunol.

[32] Lauterbach MA, Hanke JE, Serefidou M, Mangan MSJ, Kolbe CC, Hess T (2019). Toll-like receptor signaling rewires macrophage metabolism and promotes histone acetylation via ATP-citrate lyase. Immunity..

[33] Radstake TR, Roelofs MF, Jenniskens YM, Oppers-Walgreen B, van Riel PL, Barrera P (2004). Expression of toll-like receptors 2 and 4 in rheumatoid synovial tissue and regulation by proinflammatory cytokines interleukin-12 and interleukin-18 via interferon-gamma. Arthritis Rheum.

[34] Pérez-Rodríguez MJ, Ibarra-Sánchez A, Román-Figueroa A, Pérez-Severiano F, González-Espinosa C (2020). Mutant Huntingtin affects toll-like receptor 4 intracellular trafficking and cytokine production in mast cells. J Neuroinflammation.

[35] Eun-Kyu L, Sang-Mee K, Doo-Jin P, Mogg KJ, Jeehee Y (2005). Essential roles of Toll-like receptor-4 signaling in arthritis induced by type II collagen antibody and LPS. Int Immunol.

[36] Vanags D, Williams B, Johnson B, Hall S, Nash P, Taylor A (2006). Therapeutic efficacy and safety of chaperonin 10 in patients with rheumatoid arthritis: A double-blind randomised trial. Lancet..

[37] Barroso-Chinea P, Luis-Ravelo D, Fumagallo-Reading F, Castro-Hernandez J, Salas-Hernandez J, Rodriguez-Nuñez J (2019). DRD3 (dopamine receptor D3) but not DRD2 activates autophagy through MTORC1 inhibition preserving protein synthesis. Autophagy..

[38] Li Z, Ji X, Wang W, Liu J, Liang X, Wu H (2016). Ammonia induces autophagy through dopamine receptor D3 and MTOR. PLoS One.

[39] Chiamulera C, di Chio M, Cavalleri L, Venniro M, Padovani L, Collo G (2018). Ketamine effects on mammalian target of rapamycin signaling in the mouse limbic system depend on functional dopamine D3 receptors. Neuroreport..

[40] Cavalleri L, Merlo Pich E, Millan MJ, Chiamulera C, Kunath T, Spano PF (2017). Ketamine enhances structural plasticity in mouse mesencephalic and human iPSC-derived dopaminergic neurons via AMPAR-driven BDNF and mTOR signaling. Mol Psychiatry.

[41] Collo G, Cavalleri L, Bono F, Mora C, Fedele S, Invernizzi RW (2018). Ropinirole and pramipexole promote structural plasticity in human iPSC-derived dopaminergic neurons via BDNF and mTOR signaling. Neural Plast.

