# Supplementary material for: Dopamine D3 receptor signaling alleviates mouse rheumatoid arthritis by promoting Toll-like receptor 4 degradation in mast cells
Source: Cell Death Dis. 2022 Mar 15;13(3):240. doi: 10.1038/s41419-022-04695-y (PMC8924203; doi:10.1038/s41419-022-04695-y)
Supplement: Supplementary file 2 — SUPPLEMENTAL MATERIAL INFORMATION [file 41419_2022_4695_MOESM2_ESM.docx]

Dopamine D3 receptor signaling alleviates mouse rheumatoid arthritis by promoting Toll-like receptor 4 degradation in mast cells

Biao Wang^1^, Xueyi Li^2^, Ming Li^3^, Yan Geng^4^, Na Wang^5^, Yaofeng Jin^6^, Wen Zhang^7^, Ke Xu^8^, Jing Wang^9,^ Li Tao^4^, Simin Lai^1^, Kunyi Wu^5^, Jing Lei^4^, Jing Wang^1^, Ting Zhou^4^, Ke Li^5,10,^*, Yanjiong Chen^1,*^, Li Xue^4,*^

1. Department of Immunology and Pathogenic Biology, College of Basic Medicine, Xi’an Jiaotong University Health Science Center, Xi’an 710061, P. R. China;

2. Department of Rheumatology, The Second Affiliated Hospital of Xi’an Jiaotong University, Xi’an 710004, P. R. China;

3. Department of Cardiovascular Surgery, The First Affiliated Hospital of Xi’an Jiaotong University, Xi’an 710061, P. R. China;

4. Department of Clinical Laboratory, The Second Affiliated Hospital of Xi'an Jiaotong University, Xi’an 710004, P. R. China.

5. Core Research Laboratory, The Second Affiliated Hospital of Xi’an Jiaotong University, Xi’an, 710004, P. R. China;

6. Department of Pathology, The Second Affiliated Hospital of Xi’an Jiaotong University, Xi’an, 710004, P.R. China.

7. Department of Pathology, Northwest Women's and Children's Hospital, Xi'an, 710061, China.

8 Department of Joint Surgery, Xi'an Hong Hui Hospital, Xi' an Jiaotong University Health Science Center, Xi' an, 710049, P.R. China;

9 Department of Rheumatology, The First Affiliated Hospital of Xi'an Jiaotong University, Xi’an 710061, P. R. China;

10 National Local Joint Engineering Research Centre of Biodiagnostics and Biotherapy, The Second Affiliated Hospital of Xi’an Jiaotong University, Xi’an, 710004, P. R. China;

*Corresponding authors：

1. Ke Li, PhD, Core Research Laboratory, The Second Affiliated Hospital of Xi’an Jiaotong University, Email: ke.li@mail.xjtu.edu.cn;

2. Yanjiong Chen, PhD, Department of Immunology and Pathogenic Biology, College of Basic Medicine, Xi’an Jiaotong University Health Science Center, Email: chenyanjiong@mail.xjtu.edu.cn;

3. Li Xue, PhD, Department of Clinical Laboratory, The Second Affiliated Hospital of Xi'an Jiaotong University, E-mail: xuelially@163.com

**Table S1. Primers used in amplification of targeted genes.**

**Figure S1. D3R deficiency induces excessive inflammatory cytokines in the CIA model.** (A) qPCR was used to examine the mRNA levels of *il10* in mouse knee joints after CIA induction (n = 6). (B) Serum cytokines were analyzed by ELISA in mice (n = 8). All data are representative of the means ± SEM. Student’s t test was used for (A), and one-way ANOVA (Tukey’s post-hoc) was used for (B); *, p < 0.05; **, p < 0.01; ***, p < 0.001.

**Figure S2. D3R alters cytokine production in mast cells.** (A) Cytokines were tested by ELISA after BMMCs were exposed to LPS and DA (n = 8). (B) Cytokines were tested by ELISA after shRNA-treated p815 cells were exposed to LPS and DA (n = 8). LPS (100 ng/ml) and DA (1 μM) were used to activate the mast cells. All data are representative of the means ± SEM. One-way ANOVA (Tukey’s post-hoc); *, p < 0.05; **, p < 0.01; ***, p < 0.001.

**Figure S3. Ubiquitin knockdown increases cytokines in mast cells.** Cytokines were tested by ELISA after ubiquitin was silenced in BMMCs (n = 4). LPS (100 ng/ml) and DA (1 μM) were used to activate p815 mast cells. All data are representative of the means ± SEM. One-way ANOVA (Tukey’s post-hoc); *, p < 0.05; **, p < 0.01; ***, p < 0.001.

**Figure S4. Uncropped Western blots.** Frame indicate lanes that were used in figures.
