# Supplementary figures and images for: Dopamine D3 receptor signaling alleviates mouse rheumatoid arthritis by promoting Toll-like receptor 4 degradation in mast cells
Source: Cell Death Dis. 2022 Mar 15;13(3):240. doi: 10.1038/s41419-022-04695-y (PMC8924203; doi:10.1038/s41419-022-04695-y)

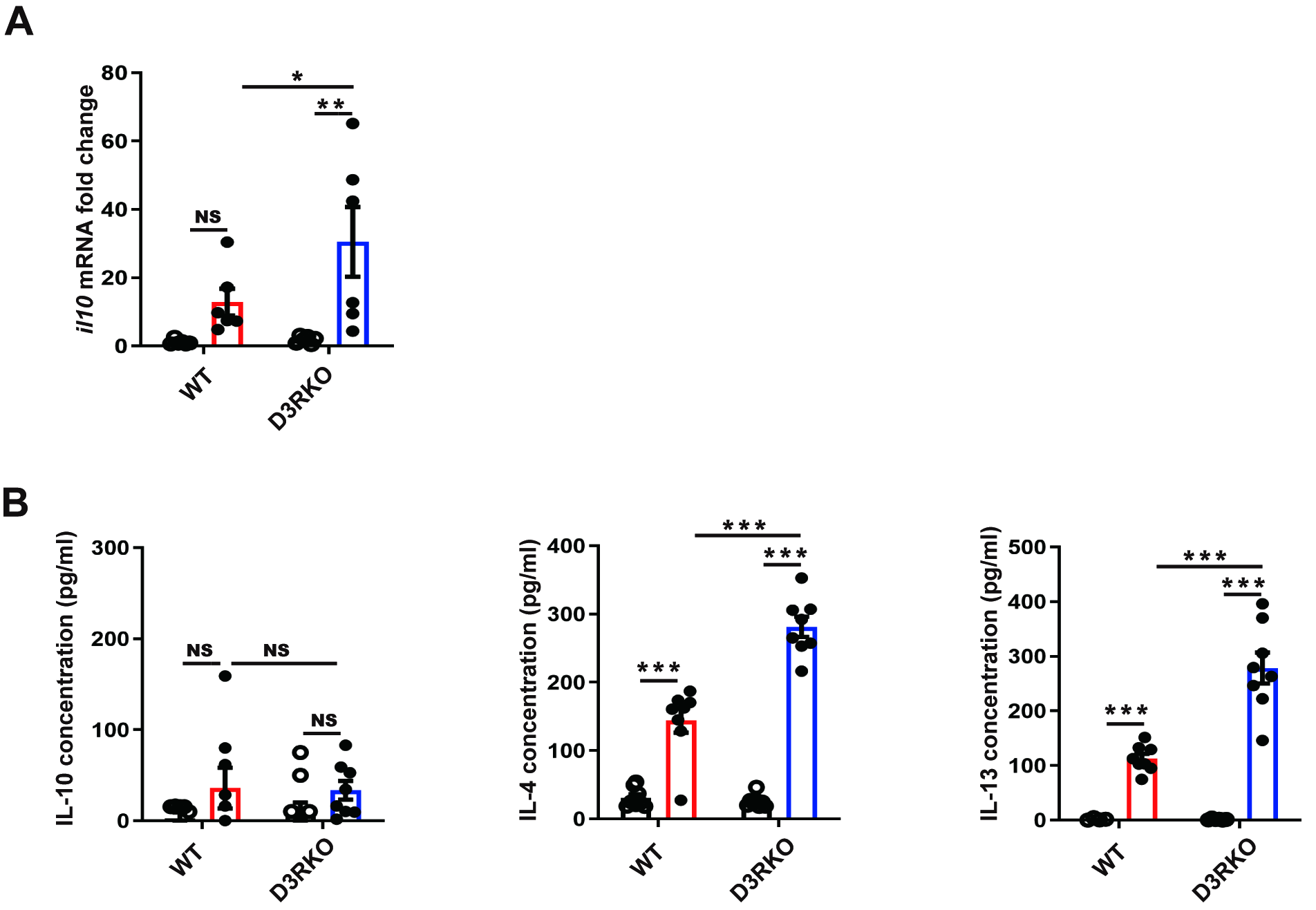

Supplement: Supplementary file 3 — Figure S1 [file 41419_2022_4695_MOESM3_ESM.tif]

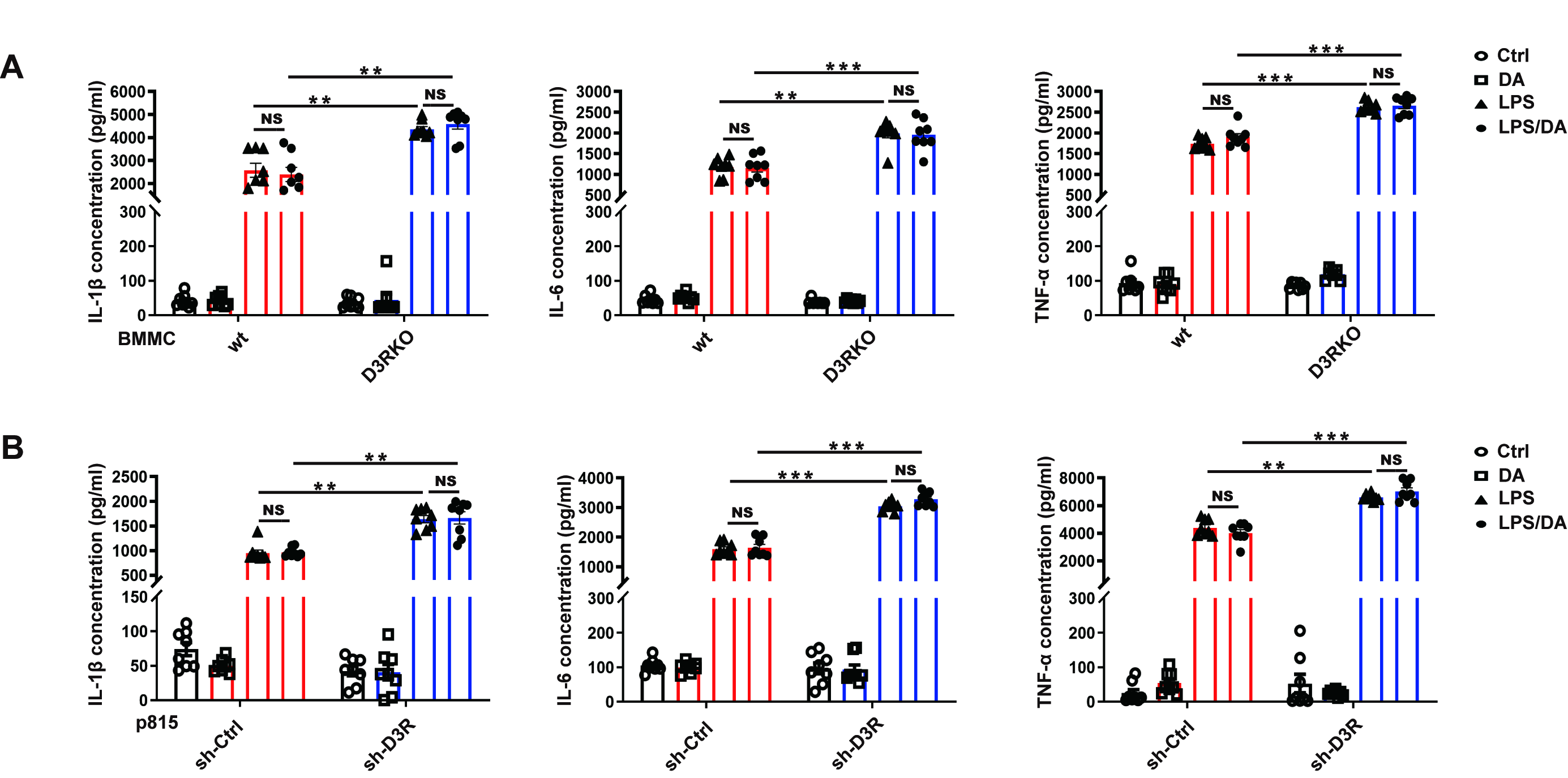

Supplement: Supplementary file 4 — Figure S2 [file 41419_2022_4695_MOESM4_ESM.tif]

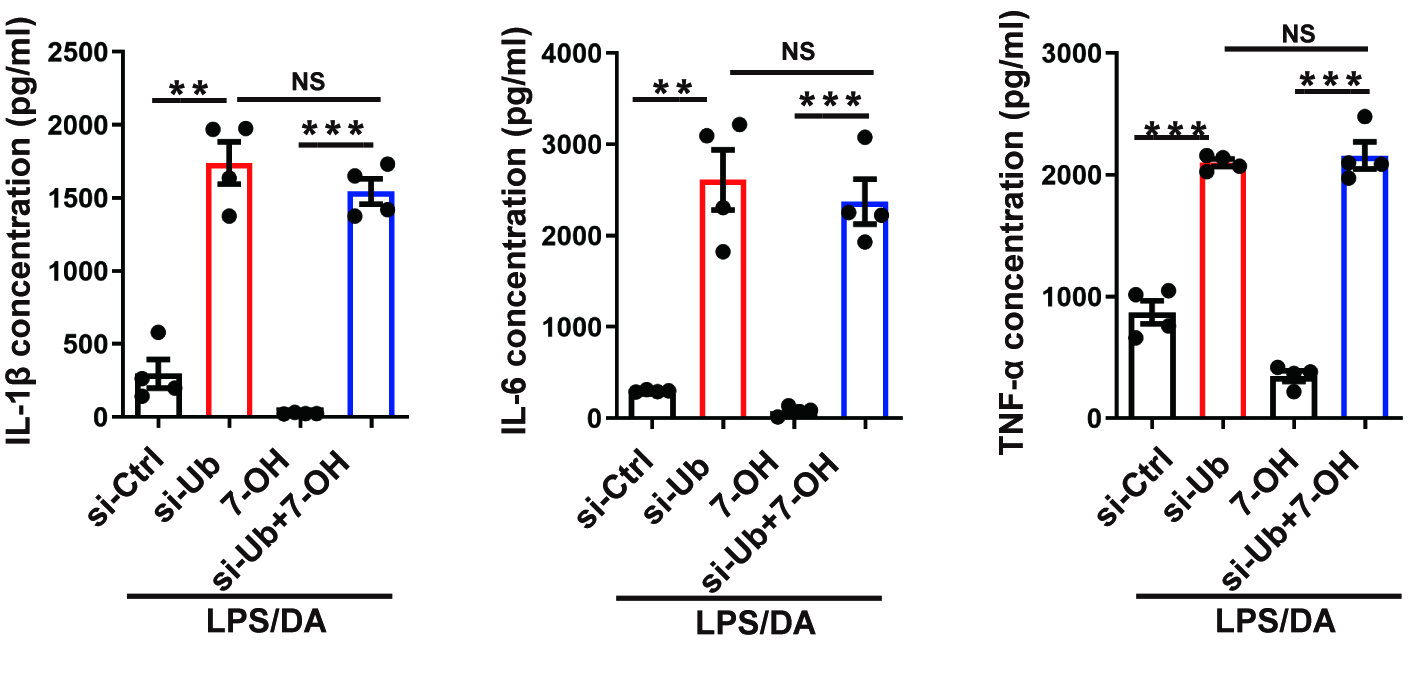

Supplement: Supplementary file 5 — Figure S3 [file 41419_2022_4695_MOESM5_ESM.tif]

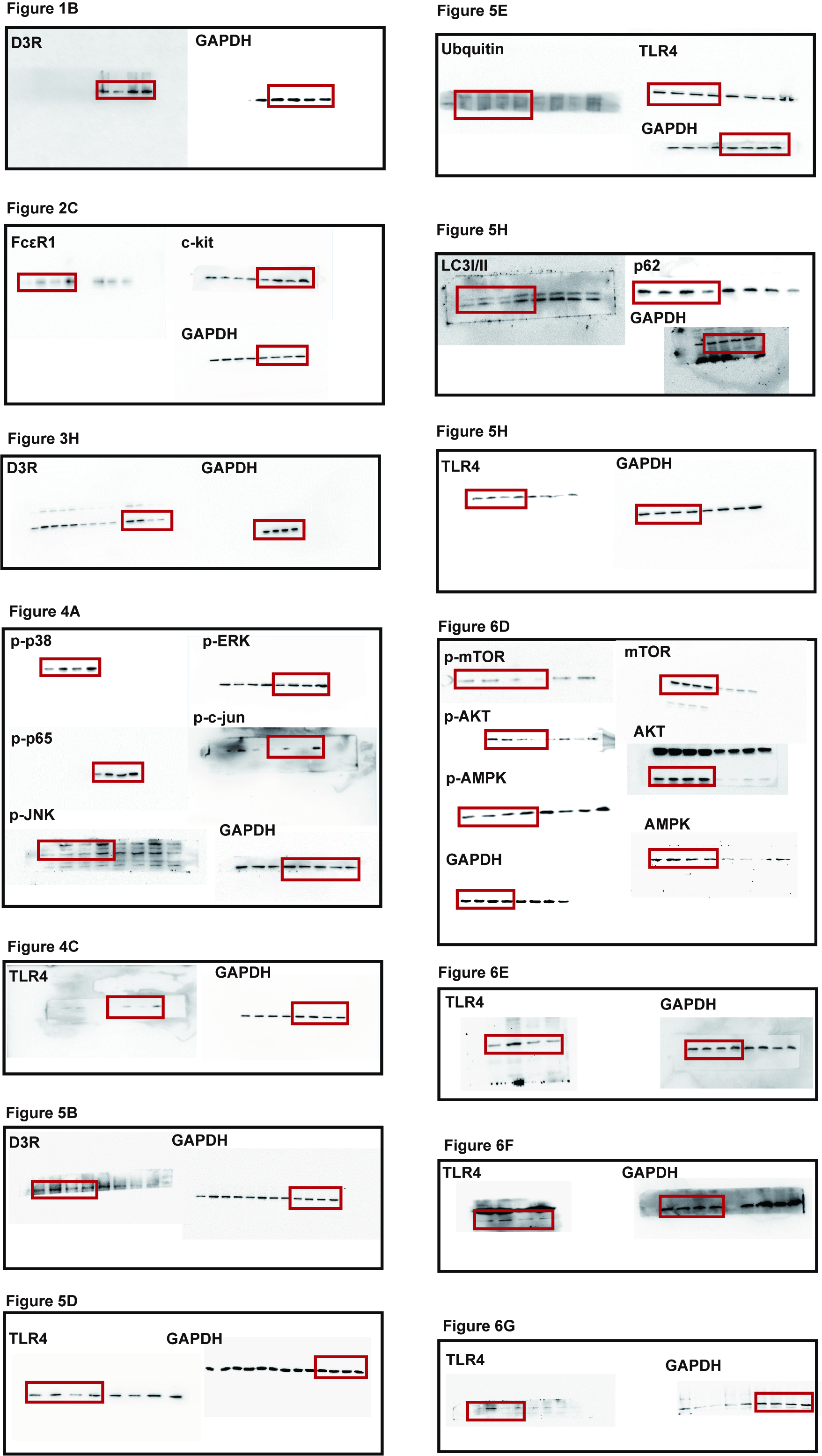

Supplement: Supplementary file 6 — Figure S4 [file 41419_2022_4695_MOESM6_ESM.tif]
