# Supplementary material for: Dopamine D3 receptor signaling alleviates mouse rheumatoid arthritis by promoting Toll-like receptor 4 degradation in mast cells
Source: Cell Death Dis. 2022 Mar 15;13(3):240. doi: 10.1038/s41419-022-04695-y (PMC8924203; doi:10.1038/s41419-022-04695-y)
Supplement: Supplementary file 7 — Table S1 [file 41419_2022_4695_MOESM7_ESM.docx]

**Table S1.** Primers used in amplification of targeted genes.

| **ID** | **Primer Sequences (5’-3’)** |
| --- | --- |
| *il6* Forward | CTGCAAGAGACTTCCATCCAGTT |
| *il6* Reverse | AGGGAAGGCCGTGGTTGT |
| *tnfa* Forward | GGCTGCCCCGACTACGT |
| *tnfa* Reverse | ACTTTCTCCTGGTATGAGATAGCAAAT |
| *il1b* Forward | GTCACAAGAAACCATGGCACAT |
| *il1b* Reverse | GCCCATCAGAGGCAAGGA |
| *gapdh* Forward | TGTGTCCGTCGTGGATCTGA |
| *gapdh* Reverse | TTGCTGTTGAAGTCGCAGGAG |
| *drd3* Forward | CTACGCCCTGTCCTACTGT |
| *drd3* Reverse | CCACCTGTCACCTCCAAG |
